# Prolonged Hypercupremia after Laparoscopic Vertical Sleeve Gastrectomy Successfully Treated with Oral Zinc

**DOI:** 10.1155/2019/8175376

**Published:** 2019-06-04

**Authors:** Timothy R. Koch, Elizabeth A. Zubowicz, John B. Gross

**Affiliations:** ^1^Center for Advanced Laparoscopic General & Bariatric Surgery, MedStar Washington Hospital Center and Georgetown University School of Medicine, Washington, DC 20010, USA; ^2^Division of Gastroenterology and Hepatology, Mayo Clinic, 200 First Street S.W., Rochester, MN 55905, USA

## Abstract

A 30-year-old female underwent vertical sleeve gastrectomy. Postoperatively, hypercupremia and elevated ceruloplasmin were identified. Further testing revealed normal blood levels of transaminases, alkaline phosphatase, and albumin. She stopped ingestion of multivitamins, began a copper-free multivitamin, and then began a low copper diet, but with no improvement in hypercupremia. Protein electrophoresis was normal with no M-spike. Urinary copper excretion was normal at 0.24 micromol/24 hours (normal: < 0.55), and there were no Kayser-Fleischer rings on slit lamp examination. Two years postoperatively, she lost 44% of excess preoperative weight and she began zinc sulfate before meals twice daily (115 mg elemental Zinc/day). At 2 months and 8 months later, plasma copper and ceruloplasmin had essentially normalized. Increased production of ceruloplasmin could have been a response to significant weight loss or the presence of nonalcoholic steatohepatitis. The mechanism of zinc's beneficial effect is uncertain but may be related to suppressing hepatic synthesis of or secretion of ceruloplasmin.

## 1. Introduction

The global epidemic of obesity continues to be a major health risk factor. The prevalence of obesity was found to have doubled since 1980 in more than 70 countries in a recent international study of 195 countries [1]. The vertical sleeve gastrectomy has become the most common operation for the surgical management of medically complicated obesity, and in 2016 a vertical sleeve gastrectomy was performed for 53.6% of 609,987 global bariatric surgical procedures [2].

Disorders of copper metabolism appear to be common after bariatric surgery [3]. In a report describing hypocupremia in individuals who had undergone Roux-en-Y gastric bypass (RYGB) surgery, Gletsu-Miller and associates also included identification of hypercupremia in 10 of 136 subjects (for a prevalence of 7.4%) [3]. In a retrospective review of 54 subjects after RYGB, we identified hypercupremia in 19% of individuals at 18 months or longer after RYGB surgery, but in only 8% of individuals at less than 18 months after RYGB surgery [4]. At that time we speculated that the likely origin for identification of hypercupremia after RYGB was the result of joint national practice guidelines recommending two multivitamins with minerals daily for postbariatric surgery patients [5], which would supply 4 mg of copper daily. We report herein a case of a woman after laparoscopic vertical sleeve gastrectomy whose hypercupremia did not improve by conversion to a copper-free multivitamin and the addition of a low copper diet.

## 2. Case Presentation

This study was reviewed and approved on December 10, 2017, by the Human Studies Subcommittee of the MedStar Research Institute, Hyattsville, MD, USA.

Micronutrient levels were measured by a commercially available private laboratory. Serum iron was determined by a colorimetric method, serum ceruloplasmin was determined by an immunological method, and plasma copper and zinc were separately determined by inductively coupled plasma/mass spectrometry.

A 30-year-old female (preoperative body mass index: 49 kg/m^2^) underwent a laparoscopic vertical sleeve gastrectomy with no immediate postoperative complication. When she was seen 5 months after surgery, she was taking two daily chewable multivitamins with minerals (containing 4 mg elemental copper and 24 mg elemental zinc). Hypercupremia was present, in association with elevated ceruloplasmin, noted in retrospect (see Table 1). Follow-up testing demonstrated persistently normal blood levels of aspartate aminotransferase, alanine transaminase, alkaline phosphatase, glucose, creatinine, free thyroxine, and albumin. She was not taking an estrogen-containing medication and she had not had placement of a copper-intrauterine device.

At 11 months after surgery, she discontinued oral intake of multivitamins. At 12 months postoperatively, when hypercupremia persisted, she began oral intake of one copper-free multivitamin daily containing 15 mg of zinc. Despite this change in her multivitamin supplementation, high levels of plasma copper persisted at her 14-month postoperative visit. A search for underlying causes showed normal protein electrophoresis (no M-spike was identified), negative celiac disease markers, and negative anti-nuclear antibody. Of note, urinary copper excretion was normal at 0.24 *μ*mol/24 hours (normal: < 0.55 *μ*mol/24 hours), which did not support the diagnosis of a copper overload disorder. She was instructed on a low-copper diet, which she then began.

At the 2-year postoperative visit, she had obtained loss of 44% of her excess preoperative weight (body mass index: 34 kg/m^2^). Hypercupremia was still present. Slit lamp examination revealed no Kayser-Fleischer rings. She then began oral zinc sulfate 220 mg taken fifteen minutes before meals twice daily (100 mg elemental Zn), with one daily copper-free multivitamin containing 15 mg zinc. At her follow-up visit 2 months later, plasma copper and serum ceruloplasmin levels had essentially normalized, and at her follow-up visit 8 months later these improvements in plasma copper and serum ceruloplasmin were reconfirmed (see Table 1).

## 3. Discussion

The differential diagnosis of hypercupremia is complex (see Table 2) and includes the following: liver disorders, gynecologic origins, endocrine origins, infections, hypertension, chronic renal failure, and neoplasia [6]. In this case report, careful examination did not identify a disorder associated with the development of hypercupremia. Individuals with persistently high blood levels of copper-binding proteins may deposit copper in Descemet's membrane and lens capsule [7]. Slit-lamp examination was however unremarkable in this patient.

Table 1 demonstrates that the high levels of plasma copper correlated with high levels of serum ceruloplasmin. At the same time, a normal urinary copper excretion and absence of Kayser-Fleischer rings excluded a more generalized copper overload syndrome. Our initial speculation was that the likely origin for the common identification of hypercupremia after bariatric surgery was the result of joint national practice guidelines recommending two multivitamins with minerals daily for postbariatric surgery patients [5]. Two daily multivitamins with minerals would supply 4 mg of copper daily. However, in our patient's case, there was no significant improvement in her hypercupremia after stopping standard multivitamin supplementation, switching to a copper-free multivitamin containing 15 mg/day zinc, or adopting a low copper diet. As seen in Table 1, hypercupremia persisted one year after stopping multivitamin supplements containing copper. In previously reported studies of patients with Wilson's disease, a low copper diet may improve clinical outcomes [8]. For these reasons, we believe that it is unlikely that the increased production of ceruloplasmin in this case was simply a reflection of an excess of available copper.

Oral zinc therapy (as zinc acetate) has been approved by the United States Food and Drug Administration for maintenance treatment of individuals with Wilson's disease who have received treatment with a chelating agent. In patients with Wilson's disease who have received chelation therapy, studies from both Europe [9] and the United States [10] have revealed that treatment with oral zinc results in identification of increased liver copper concentrations at follow-up liver biopsies.

**Figure 1:**
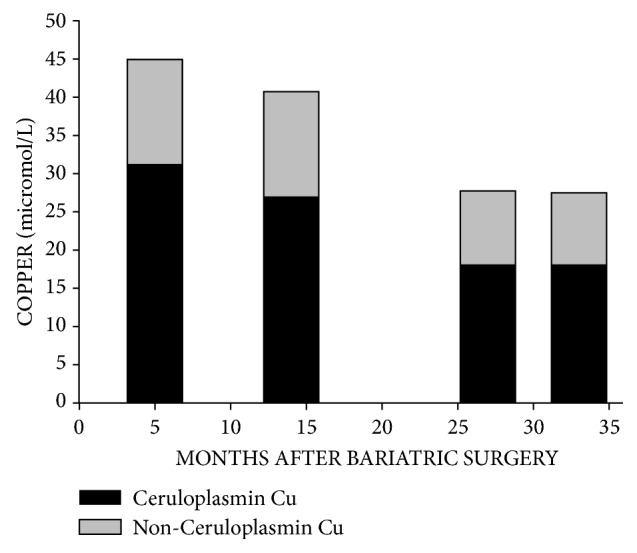
Concentrations of plasma copper (Cu) are shown at postoperative visits. The black bars demonstrate the contributions of ceruloplasmin copper to the plasma copper concentrations while the gray bars demonstrate the contributions of non-ceruloplasmin copper to the plasma copper concentrations. Note that there was a 38% decline in plasma copper between the 5-month postoperative visit and the postoperative visit after initiation of therapy with oral zinc.

One possible interpretation of these results is that oral zinc suppresses hepatic synthesis of or secretion of ceruloplasmin. To examine this possible mechanism, in Figure 1, we depict postoperative copper levels related to ceruloplasmin copper and non-ceruloplasmin copper. In examination of hypercupremia at the 5-month postoperative visit compared to the plasma copper concentration after initiation of oral zinc, Figure 1 demonstrates the 38% decline in plasma copper. Within this decline, there was a 42% decline in ceruloplasmin copper, but a 29% decline in non-ceruloplasmin copper. This finding could support the notion that oral zinc suppresses hepatic synthesis of or secretion of ceruloplasmin. Additional studies utilizing serial liver biopsies for determination of liver copper and ceruloplasmin concentrations are required to examine this potential mechanism.

Increased production of ceruloplasmin in this patient could have been a response to significant weight loss or to the presence of nonalcoholic fatty liver disease, since her body mass index was still 34 kg/m^2^ in follow-up after bariatric surgery. An initial report described an association between nonalcoholic fatty liver disease and low liver copper concentrations [11]. In that report, lower liver copper was reported in those individuals with more severe hepatic steatosis [11], which raises the question of whether the finding of low liver copper was a “dilution” effect due to the presence of extensive steatosis. A recent review of the potential role of copper in nonalcoholic fatty liver disease suggested that serum copper levels rise in the progression from nonalcoholic fatty liver disease to nonalcoholic steatohepatitis and that antioxidant food agents that bind copper may be useful in the treatment of nonalcoholic fatty liver disease [12]. Since serum ceruloplasmin may be a reactive inflammatory marker, a cautious interpretation of the reported rise in serum copper levels [12] may be justified.

In conclusion, hypercupremia may occur after vertical sleeve gastrectomy and after RYGB. This finding stands in contrast to reports of hypocupremia after RYGB [3, 4]. Use of a daily copper-free multivitamin with mineral supplement and instruction on a low copper diet are reasonable initial approaches for the treatment of hypercupremia. In individuals with persistent hypercupremia, an evaluation for conditions associated with hypercupremia is important and should include testing for monoclonal gammopathy and slit lamp examination. This report suggests that oral zinc may be effective in decreasing the concentrations of serum ceruloplasmin and plasma copper in patients with hypercupremia following vertical sleeve gastrectomy.

## Figures and Tables

**Table 1:** Summary of biochemical findings.

Months after surgery	Copper	Ceruloplasmin	Zinc	Iron
5 months	45	4.9	14	10
12 months*∗*	41		12	
14 months†	41	4.8		
17 months‡	36			
24 months	39			16
27 months¥	28	2.9	12	
33 months*∗∗*	28	2.9	15	
	(Normal range: 11-26 *µ*mol/L)	(Normal range: 1.4-2.9 *µ*mol/L)	(Normal range: 8.6-21 *µ*mol/L)	(Normal range: 4.8-28 *µ*mol/L)

*∗*Multivitamin had been stopped.

†Taking a copper-free multivitamin containing 15 mg/day zinc.

‡Using a low copper diet and taking a copper-free multivitamin with 15 mg/day zinc.

¥Two months after starting 115 mg/day zinc; *∗∗*eight months after starting 115 mg/day zinc.

**Table 2:** Differential diagnosis of hypercupremia.

PBC*∗*	PSC*∗∗*
Cirrhosis	Hemochromatosis
Pregnancy	Oral contraceptive/estrogen use
Thyrotoxicosis	Diabetes mellitus
Hypertension	Infections
Chronic renal failure	
Lung & breast cancer	Leukemia
Monoclonal gammopathy	

*∗*PBC: primary biliary cholangitis; *∗∗*PSC: primary sclerosing cholangitis.
